# Cytoplasmic Localization of WT1 and Decrease of miRNA-16-1 in Nephrotic Syndrome

**DOI:** 10.1155/2017/9531074

**Published:** 2017-02-19

**Authors:** Pablo Zapata-Benavides, Mariela Arellano-Rodríguez, Juan José Bollain-y-Goytia, Moisés Armides Franco-Molina, Gloria Azucena Rangel-Ochoa, Esperanza Avalos-Díaz, Rafael Herrera-Esparza, Cristina Rodríguez-Padilla

**Affiliations:** ^1^Departamento de Microbiología e Inmunología, Facultad de Ciencias Biológicas, Universidad Autónoma de Nuevo León (UANL), 66450 San Nicolás de los Garza, NL, Mexico; ^2^Laboratorios de Inmunología y Biología Molecular, Unidad Académica de Ciencias Biológicas, Universidad Autónoma de Zacatecas (UAZ), 98040 Zacatecas, ZAC, Mexico; ^3^Hospital General Zacatecas “Luz González Cosío”, Ciudad Gobierno, 98160 Zacatecas, ZAC, Mexico

## Abstract

Nephrotic syndrome (NS) is a glomerular disease that is defined by the leakage of protein into the urine and is associated with hypoalbuminemia, hyperlipidemia, and edema. Steroid-resistant NS (SRNS) patients do not respond to treatment with corticosteroids and show decreased Wilms tumor 1 (WT1) expression in podocytes. Downregulation of WT1 has been shown to be affected by certain microRNAs (miRNAs). Twenty-one patients with idiopathic NS (68.75% were SSNS and 31.25% SRNS) and 10 healthy controls were enrolled in the study. Podocyte number and WT1 location were determined by immunofluorescence, and the serum levels of miR-15a, miR-16-1, and miR-193a were quantified by RT-qPCR. Low expression and delocalization of WT1 protein from the nucleus to the cytoplasm were found in kidney biopsies of patients with SRNS and both nuclear and cytoplasmic localization were found in steroid-sensitive NS (SSNS) patients. In sera from NS patients, low expression levels of miR-15a and miR-16-1 were found compared with healthy controls, but only the miR-16-1 expression levels showed statistically significant decrease (*p* = 0.019). The miR-193a expression levels only slightly increased in NS patients. We concluded that low expression and delocalization from the WT1 protein in NS patients contribute to loss of podocytes while modulation from WT1 protein is not associated with the miRNAs analyzed in sera from the patients.

## 1. Introduction

Nephrotic syndrome (NS) is one of the most common diseases in children and is characterized by the leakage of large amounts of protein into the urine, lipidemia, hypoalbuminemia, and dysfunction of the glomerular filtration barrier [1]; NS is also a major cause of podocyte injury [2]. Podocytes, highly specialized cells of the visceral epithelium, are found in the glomerular membrane of the kidney [3]; they constitute the terminal part of the ultrafiltration barrier that prevents protein loss, which can result in damage and detachment of podocytes. The reduction of glomerular podocytes in lupus nephritis (LN) patients is correlated with the cumulative loss of urinary podocytes and proteinuria during the progression of kidney disease [2, 4].

Some urinary biomarkers have been found to be associated with injured podocytes in urine sediment, such as podocalyxin (PODXL), nephrin (NPHS1), podocin, and Wilms tumor protein (WT1) [5]. WT1 itself has been found in exosomes in urine sediment, indicating podocyte injury [6].

WT1 is essential for urogenital development, and its expression is limited to mature podocytes [7]. The* WT1* gene encodes for a transcription factor that is critical for the development of podocytes and viability; the regulation of podocyte homeostasis occurs via PODXL and NPHS1 [7].

Recent studies have demonstrated that mutations in* WT1* can lead to syndromic forms of steroid-resistant NS (SRNS), such as Denys-Drash or Frasier syndrome, and can cause isolated SRNS [8, 9]. Between 10% and 20% of patients fail to respond to steroid therapy; thus, the prognosis for SRNS is usually poor, due to the increased risk of developing end-stage renal disease [10, 11]. Beltcheva et al. reported a case of a pediatric patient with SRNS that was caused by a novel dominant* WT1* mutation, C11184A, identified in exon 9 [10]. Mutations within* WT1* are frequent causes of sporadic, isolated cases of SRNS in girls [8].* WT1* expression is modulated by microRNAs (miRNAs) [12], which are small (20–25 nucleotides), noncoding RNA molecules that act as posttranscriptional regulators of gene expression in animals and plants, participating in many key biological processes [13]. In the kidney, miRNAs play critical roles in renal development, homeostasis, and physiological function. Several studies have shown that miRNAs are also key mediators of the pathogenesis of various renal diseases [14–16]. Furthermore, miRNAs can be secreted into the extracellular environment and detected in fluids, including urine and serum. They have been proposed as biomarkers for a wide range of diseases [17, 18].

Gao et al. discovered that miRNAs 15a and 16-1 may function as tumor suppressors to regulate leukemic cell proliferation, potentially by downregulating the* WT1 *oncogene [12]; pure curcumin was also recently shown to modulate the expression of WT1, partly by upregulating the expression of miR-15a and miR-16-1 in leukemic cells [19].

In the podocytes of patients with focal segmental glomerulosclerosis (FSGS), the expression of miR-193a was induced, which in turn inhibited the expression of* WT1*, modulating the expression of genes critical for podocyte architecture [20]. The presence of miRNAs in plasma, serum, and urine was also shown to be a possible biomarker of diabetic kidney disease [21]. Another mechanism of modulation of* WT1* that has been observed in experimental mice during the sepsis is the decrease of nuclear WT1 in podocytes and was associated with transcriptional suppression of nephrin and cause albuminuria [22].

The aim of this study was to determine whether there are different expression levels and protein localization of WT1 in SRNS and SSNS patients and its relationship with the miR-15a, miR-16-1, and miR-193a, which modulate the* WT1* expression in other models.

## 2. Methods

Twenty-one biopsies, serum and urine samples from 11 males and 10 females, from patients diagnosed with NS who met the criteria of International Study of Kidney Disease in Children with proteinuria levels greater than 3.5 g/24 h were included, in the control group serum and urine samples were obtained from 10 subjects without kidney disease, renal biopsies were obtained of autopsy subjects without renal disease who died in accidents as a result of a head injury. Patients who agreed to participate in the study signed a letter of informed consent. This study was performed with patients living in the north-central region from Mexico, according to the principles of the Declaration of Helsinki, and was approved by the ethics committees of our institutions.

### 2.1. Sample Processing

Freshly urine samples were processed in less than 30 min, and 10 mL of urine was centrifuged for 5 min at 1,500 rpm. After decanting the supernatant, the pellet was resuspended in 1 mL PBS-TS (0.1% Tween-20 and 0.02% SDS in phosphate-buffered saline [PBS]); 25 *µ*L of which was fixed at 55°C for 10 min for continued permeabilization as described below.

### 2.2. Immunofluorescence

As the localization of WT1 is essential for its biological action, WT1 localization was assessed for all samples. For the analysis of the number of podocytes per glomerulus in both NS patients and healthy subjects, WT1 and nephrin were used as biomarkers for immunofluorescence of 4 *μ*m thick sections of renal tissue mounted on microscope slides. The specimens were dewaxed, permeabilized with Triton X-100 (Triton 0.1% with 1% sodium citrate in PBS), and washed three times with PBS. The tissues were blocked with 20% fetal bovine serum (FBS) in PBS for 30 min and incubated for 1 h with monoclonal anti-WT1 antibody (WT1 [F-6] sc-7585, monoclonal mouse IgG1, Santa Cruz Biotechnology, Santa Cruz, CA, USA) or anti-nephrin monoclonal antibody (H-300 sc-28192, polyclonal rabbit IgG, Santa Cruz Biotechnology) diluted 1 : 100 in 10% FBS in PBS. After washes with PBS, the presence of bound antibody was identified by goat anti-mouse IgG1 Texas Red (sc-2979, Santa Cruz Biotechnology) and goat anti-rabbit IgG-FITC (sc-2012, Santa Cruz Biotechnology) staining. Some slides were counterstained with 4′,6-diamidino-2-phenylindole (DAPI). Finally, the slides were mounted and examined by confocal scanning microscopy. The intensity of the signal was expressed in pixels and analyzed using Image-Pro Plus software, version 7.0 (Media Cybernetics, Rockville, MD, USA). The WT1 localization was determined by Pearson correlation coefficient (PCC) [23].

### 2.3. Blood Collection

Five mL whole blood samples from NS patients and healthy controls were collected in tubes (BD Vacutainer; Becton Dickinson Vacutainer Systems, Franklin Lakes, New Jersey, USA) and then processed for obtain the serum by centrifugation at 1,500 rpm for 10 min. The sera were stored at −70°C until analysis.

### 2.4. RNA Isolation and Quantification

Total RNA was extracted from patient sera using the miRNeasy Serum/Plasma Kit (QIAGEN, Hilden, Germany), according to the manufacturer's instructions. Two aliquots of 200 *µ*L each were used to obtain sufficient RNA to perform all quality tests. RNA was dissolved in diethylpyrocarbonate-treated water, and RNA quality and quantity were determined by spectrophotometry using the NanoDrop ND-1000 (NanoDrop Technologies, Wilmington, DE, USA).

### 2.5. cDNA Synthesis and Real Time RT-PCR (qPCR) Assay for miRNAs Expression

To determine whether miR-15a, miR-16-1, and miR-193a are involved in regulating the expression of* WT1*, their expression was quantified in sera from NS patients and healthy controls. Total RNA was polyadenylated and subjected to reverse transcription using an NCode miRNA First-Strand cDNA Synthesis Kit (Invitrogen Carlsbad, CA) according to the manufacturer's instructions.

Real-time qPCR analysis was carried out using SYBR® Green PCR Master Mix (Applied Biosystems; Foster City, CA, USA) on an ABI 7500 Fast Real-Time PCR System (Applied Biosystems). The primers reported by Gao et al. [12] were used to quantify the relative expression levels of miR-15a, miR-16-1, and miR-193a in the samples. Experiments were performed in triplicate. U6 was used as an endogenous control. The expression of each miRNA relative to U6 RNA was calculated using the equation 2^−ΔΔCT^ [24–26].

### 2.6. RNA Isolation and qPCR for* WT1* mRNA Expression

Total RNA was isolated from renal biopsies using the High Pure RNA Isolation Kit and High Pure RNA Paraffin Kit (Roche Life Science, Mexico) according to the manufacturer's instructions. Single-stranded cDNA was synthesized by reverse transcription using the miScript Reverse Transcription Kit (Qiagen, United States) according to the manufacturer's instructions. Real-time PCR was performed using the ABI 7500 Fast Real-Time PCR System (Applied Biosystems, Foster City, CA, USA) with TaqMan gene expression assays. Comparative real-time PCR assays were performed for each sample in triplicate. The primers for WT1-F: 5′-TCTGCGGAGCCCAATACAG-3′; WT1-R: 5′-CACATCCTGAATGCCTCTGAAGA-3′; and WT1-P FAM: 5′-CACCGTGCGTGTGTATT-TAMRA-3′ were used. The comparative quantification cycle threshold (C_q_) method was used to determine the relative expression levels of the* WT1* gene. The* 18S rRNA* (Applied Biosystems, Foster City, CA, USA) gene was used for normalization.

### 2.7. Statistical Analyses

Statistical analyses were performed using SPSS 17.0 software. The results are presented as the mean  ±  SD. Comparisons were made using an analysis of variance and Student's *t*-test, and the association between miRNA levels and expression of* WT1* was assessed by Pearson's correlation statistical program and considered significant at* p* value less than or equal to 0.05.

## 3. Results

### 3.1. Sample Description

Twenty-one samples from patients with NS and 10 healthy subjects without renal disease as control group (Table 1) were analyzed for levels of creatinine (0.39 ± 0.25 mg/dL), proteinuria (4.01 ± 2.7 g/24 h), cholesterol (207.06 ± 91.72 mg/dL), triglycerides (141.56 ± 93.55 mg/dL), and albumin (3.75 ± 0.83 g/dL). Control renal biopsies were obtained from autopsy subjects without renal disease who died in accidents as a result of a head injury.

### 3.2. WT1 Localization in Podocytes in NS Patients and Healthy Subjects

**Figure 1:**
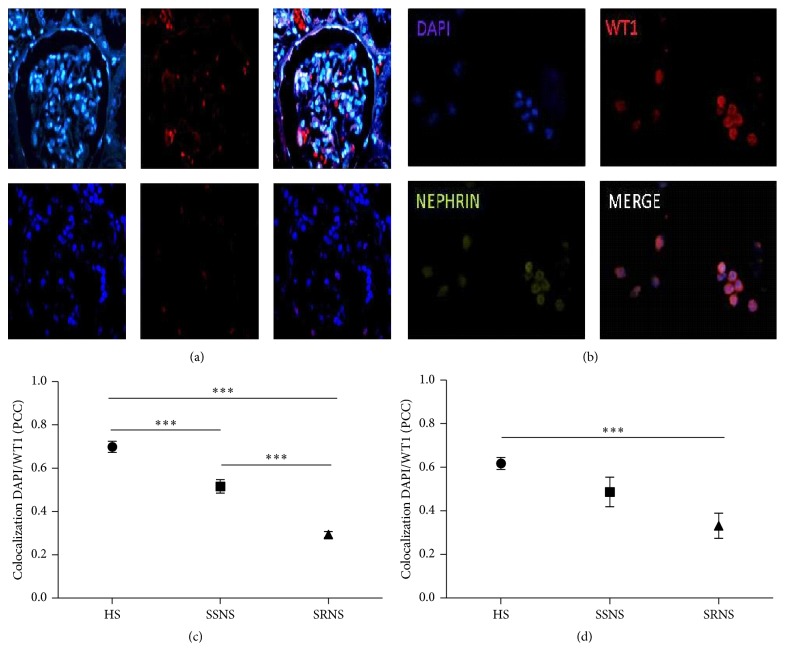
WT1 expression and localization in podocytes from biopsies and urinary sediments of patients with NS. (a) Immunofluorescence of renal biopsies (magnification, ×40) of patients with nephrotic syndrome (NS). (b) Immunofluorescence of podocytes in urine sediments: nephrin detection was used as a second marker of podocytes (magnification, ×40). (c) Colocalization of DAPI and Wilms tumor 1 (WT1) in nucleus/cytoplasm of podocytes in kidney biopsies. (d) Colocalization of DAPI and WT1 in nucleus/cytoplasm of podocytes in urinary sediments. The colocalization was determined by Pearson correlation coefficient (PCC). ^*∗*^*p* < 0.05, ^*∗∗*^*p* < 0.01, and ^*∗∗∗*^*p* < 0.001.

Immunofluorescence of renal biopsy tissue using WT1 as a biomarker showed a decrease of 27% in NS patients compared to healthy subjects (Figure 1(a)). The localization of WT1 in SRNS patients was primarily cytoplasmic, whereas WT1 in SSNS patients showed both a nuclear and a cytoplasmic localization while in healthy controls it was mainly nuclear. The localization assay was performed by the colocalization of DAPI (nuclear localization) and Texas Red (WT1), showing a statistically significant difference between SSNS (PPC = 0.51 ± 0.03) and SRNS (PPC = 0.29 ± 0.01) patients compared with healthy subjects (PPC = 0.73 ± 0.02) (*p* = 0.00001) (Figure 1(c)). In urine sediments of SRNS patients, detached podocytes showed a cytoplasmic localization of WT1 (PPC = 0.35 ± 0.08) (Figure 1(b)). These results were significantly different from those in healthy subjects (PPC = 0.64 ± 0.03) (*p* ≤ 0.0001; Figure 1(d)).

### 3.3. Analysis of* WT1* Expression in Podocytes of NS Patients and Healthy Controls

**Figure 2:**
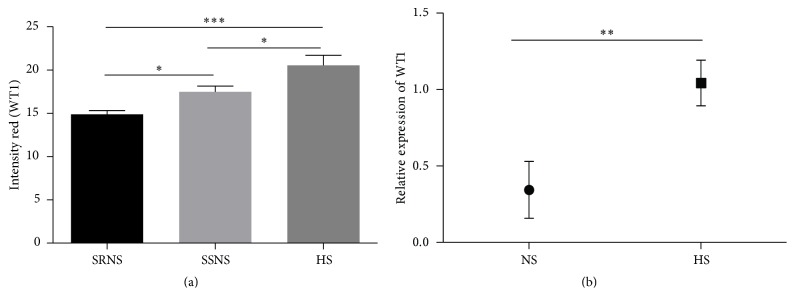
WT1 expression in podocytes from renal biopsies of patients with NS. (a) WT1 expression immunofluorescence. HS: healthy subjects; SSNS: steroid-sensitive NS; and SRNS: steroid-resistant NS. WT1 expression was measured by pixel intensity. (b) Relative expression levels of WT1 by real-time PCR. ^*∗*^*p* < 0.05, ^*∗∗*^*p* < 0.01, and ^*∗∗∗*^*p* < 0.001.

*WT1* expression analysis in podocytes was performed by two methods: immunofluorescence and RT-qPCR.* WT1* expression by intensity pixels in renal biopsies of patients with NS was statistically different than healthy controls (*p* = 0.0001) and between patients with SSNS and SRNS (*p* = 0.05; Figure 2(a)), with highest levels in healthy controls and lowest levels in SRNS patients.* WT1* RNA expression levels were analyzed by RT-qPCR in renal biopsies of NS patients and healthy controls, showing significantly lower (66%) WT1 mRNA levels in NS patients (*p* = 0.017), indicating a decrease in basal* WT1* levels in the podocytes of NS patients (Figure 2(b)).

### 3.4. Expression of miR-15a, miR-16-1, and miR-193a Levels in Sera from NS Patients

**Figure 3:**
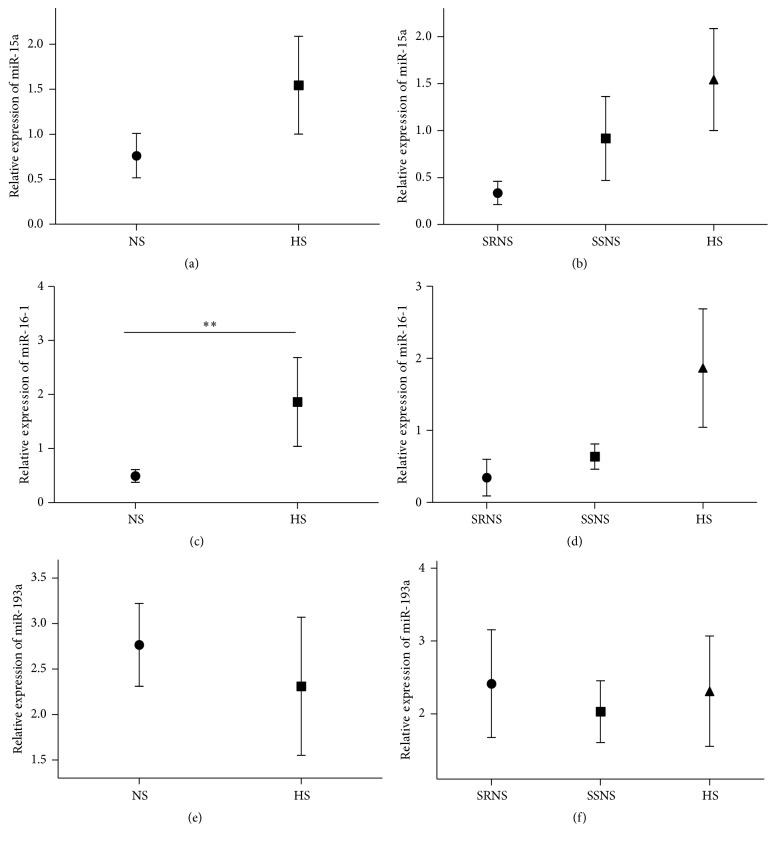
Expression of miR-15a, miR-16-1, and miR-193a in sera from patients with NS. Relative expression levels of miR-15a (a) in patients with NS (nephrotic syndrome) and healthy subjects (HS) and (b) in patients with steroid-sensitive NS (SSNS) and steroid-resistant NS (SRNS). miR-16-1 (c) in patients with NS and HS and (d) in patients with SSNS and SRNS and miR-193a (e) in patients with NS and HS and (f) in patients with SSNS and SRNS.^*∗*^*p* < 0.05, ^*∗∗*^*p* < 0.01, and ^*∗∗∗*^*p* < 0.001.

The expression levels of miR-15a (2^−ΔΔCT^) did not differ between the different groups (SRNS 0.33 ± 0.12, SSNS 0.91 ± 0.44, and HS 1.54 ± 0.54 of relative expression) (*p* = 0.138; Figure 3(a)) or between patients with SRNS and SSNS compared to the control group (*p* = 0.31; Figure 3(b)). While the expression of miR-16-1 was significantly lower (0.54 ± 0.144 relative expression) in the sera of NS patients compared with healthy controls (1.86 ± 0.82 relative expression) (*p* = 0.019; Figure 3(c)), there was no difference between SSNS and SRNS patients (*p* = 0.14; Figure 3(d)). The relative expression of miR-193 (2^−ΔΔCT^) did not differ between NS patients (2.14 ± 0.36 relative expression) and controls (2.30 ± 0.75 relative expression,* p* = 0.59; Figure 3(e)) or between patients with SRNS and SSNS (*p* = 0.91; Figure 3(f)).

### 3.5. Relationship between WT1 and miRNAs

We did not find a significant correlation between WT1 expression and serum concentrations of miR-15a, miR-16-1, and miR-193a in NS patients (miR-15a: *r* = 0.118,* p* = 0.882; miR-16-1: *r* = 0.301,* p* = 0.969; and miR-193a: *r* = 0.213,* p* = 0.787). However, a positive correlation was found between miR-15a and miR-16-1 (*r* = 0.994,* p* = 0.006). There was no significant relationship between the particular miRNA levels.

## 4. Discussion

Epidemiological studies indicate that NS remains the most common manifestation of glomerular disease in childhood, often causing death from untreated infections [27]. The present study reviewed 21 children with NS in the Mexican mestizo population, with an average age of 5.8 years.

Idiopathic NS is one of the most common glomerular diseases in pediatrics. The response to steroids is the best prognostic factor in this disease, and most children with NS respond to corticosteroids. However, 70% of children experience recurrence, with episodes of edema and proteinuria [28]; fewer than 3% of patients with SSNS progress to chronic renal failure compared to 50% patients with SRNS [29].

The WT1 protein has been used as a biomarker of podocyte loss from glomeruli in kidney disease [30, 31] and of the presence of damaged podocytes in urinary sediment [32, 33]. In this work, we clearly observed a reduction of podocytes in renal biopsies of children with NS and the presence of podocytes in the urine, but the reason for such podocyte detachment is not clear. The cause may be an imbalance between different isoforms of WT1 or decreased basal protein expression. Different research groups have observed a decrease in WT1 protein expression in children with SRNS, which was often accompanied by* WT1* mutations in exons 8 and 9 [8, 34, 35]. However, another report suggested that reduced expression levels of WT1 cause glomerulonephritis and mesangial sclerosis, depending on the level of the protein [36].

We found normal WT1 expression in the nuclei of podocytes from biopsies of healthy controls without kidney disease; however, in SSNS patients, WT1 expression was localized to both the nucleus and cytoplasm, whereas WT1 expression is primarily cytoplasmic in SRNS patients, suggesting that delocalization of WT1 from the nucleus can lead to a loss of function as a transcription factor. Decreased expression levels of WT1 lead to downregulation of its target genes* PODXL* (podocalyxin) and* NPHS1* (nephrin), as well as several other genes crucial for the architecture of podocytes, initiating a catastrophic collapse of the entire podocyte-stabilizing system [20]. Additionally, Kato et al. [22] observed that in Lipopolysaccharide (LPS) treated mice, a loss of albumin decreased nephrin levels and the nuclear localization of WT1 in podocytes. The nuclear localization of WT1, as well as nephrin mRNA and protein levels, returned to near basal levels 72 hours after LPS with recovery of albumin levels [22].

The loss of* WT1* gene expression could be a fatal consequence as described above [9]; the regulation of* WT1* is provided by certain genes, such as* PAX2* and* PAX8*, or epigenetic modulation mechanisms, such as miRNAs [12, 19]. In addition,* WT1* has the ability to self-regulate [37]. In this study, we assessed whether low WT1 expression in podocytes correlates with the circulating levels of miR-15a, miR-16-1, and miR-193a in the sera of NS patients. Our results indicate that the expression of miR-15a was higher in healthy controls than in NS patients but was not statistically significant. Similar results were observed for miR-16-1, but the difference between the expression of miR-16-1 in NS patients and in healthy controls was significant, indicating an inverse relationship between miRNAs and WT1 expression. Gao et al. reported that miR-15a and miR-16-1 inhibit the proliferation of leukemic cells by decreasing WT1 levels via joining* WT1*-3′UTR. The expression of these miRNAs inhibits cell proliferation, promotes apoptosis of cancer cells, and suppresses tumorigenicity both in vitro and in vivo [12]. Both miR-15a and miR-16-1 have been shown to negatively affect several oncogenes, including* BCL2*,* MCL1*, C*CND1*, and* WNT3A*. Downregulation of these miRNAs has been reported in chronic lymphocytic lymphoma, pituitary adenoma, and prostatic carcinoma [37, 38], while miR-193a was not found to elicit any significant effects on these cancers. However, the data reported by Gebeshuber et al. [20] indicated that miR-193a is involved in FSGS by decreasing WT1 levels. In conclusion, we found low WT1 expression and delocalization in NS patients. We also observed higher expression levels of miR-15a and miR-16-1 in healthy controls compared with NS patients, but only miR-16-1 levels were statistically different. However, we did not find a correlation between miRNA expression and WT1 levels. It is important to extend the search for miRNAs as biomarkers of renal diseases as well as to understand the basic mechanisms of gene regulation of podocytes and the glomerular filtration system.

## Figures and Tables

**Table 1:** Description of NS patients and controls.

	Age	Gender	%	Treatment	%	Proteinuria
Nephrotic syndrome	5.58 (range 2–12 years)	Males	52	SSNS	68.75	4.01 g/24 h
		Females	48	SRNS	31.25	
Healthy subjects	9 (range 3–12 years)	Males	30.77	No treatment		<0.3 g/L
		Females	69.23			

SSNS: steroid-sensitive nephrotic syndrome; SRNS: steroid-resistant nephrotic syndrome.

## References

[1] Macé C., Chugh S. S. (2014). Nephrotic syndrome: components, connections, and angiopoietin-like 4-related therapeutics. *Journal of the American Society of Nephrology*.

[2] Bierzynska A., Soderquest K., Koziell A. (2014). Genes and podocytes—new insights into mechanisms of podocytopathy. *Frontiers in Endocrinology*.

[3] Lasagni L., Romagnani P. (2013). Basic research: podocyte progenitors and ectopic podocytes. *Nature Reviews Nephrology*.

[4] Sinha A., Bagga A. (2012). Nephrotic syndrome. *Indian Journal of Pediatrics*.

[5] Sekulic M., Pichler Sekulic S. (2013). A compendium of urinary biomarkers indicative of glomerular podocytopathy. *Pathology Research International*.

[6] Zhou H., Kajiyama H., Tsuji T. (2013). Urinary exosomal wilms' tumor-1 as a potential biomarker for podocyte injury. *American Journal of Physiology - Renal Physiology*.

[7] Bariety J., Mandet C., Hill G. S., Bruneval P. (2006). Parietal podocytes in normal human glomeruli. *Journal of the American Society of Nephrology*.

[8] Yang Y. H., Zhao F., Feng D. N. (2013). Wilms' tumor suppressor gene mutations in girls with sporadic isolated steroid-resistant nephrotic syndrome. *Genetics and Molecular Research*.

[9] Feng D. N., Yang Y. H., Wang D. J. (2014). Mutational analysis of podocyte genes in children with sporadic steroid-resistant nephrotic syndrome. *Genetics and Molecular Research*.

[10] Beltcheva O., Boueva A., Morgunova E. (2011). Novel mutation in Wilms' tumour 1 gene associated with steroid-resistant nephrotic syndrome. *NDT Plus*.

[11] Mucha B., Ozaltin F., Hinkes B. G. (2006). Mutations in the Wilms' tumor 1 gene cause isolated steroid resistant nephrotic syndrome and occur in exons 8 and 9. *Pediatric Research*.

[12] Gao S.-M., Xing C.-Y., Chen C.-Q., Lin S.-S., Dong P.-H., Yu F.-J. (2011). MiR-15a and miR-16-1 inhibit the proliferation of leukemic cells by down-regulating WT1 protein level. *Journal of Experimental & Clinical Cancer Research*.

[13] Hou J., Zhao D. (2013). MicroRNA regulation in renal pathophysiology. *International Journal of Molecular Sciences*.

[14] Chung A. C.-K., Lan H. Y. (2015). MicroRNAs in renal fibrosis. *Frontiers in Physiology*.

[15] Wang H., Hu Z., Chen L. (2015). Decreased serum miR-503 level in children with nephrotic syndrome. *Clinical Laboratory*.

[16] Rudnicki M., Perco P., D'haene B. (2016). Renal microRNA- and RNA-profiles in progressive chronic kidney disease. *European Journal of Clinical Investigation*.

[17] Tiberio P., Callari M., Angeloni V., Daidone M. G., Appierto V. (2015). Challenges in using circulating miRNAs as cancer biomarkers. *BioMed Research International*.

[18] Ramezani A., Devaney J. M., Cohen S. (2015). Circulating and urinary microRNA profile in focal segmental glomerulosclerosis: a pilot study. *European Journal of Clinical Investigation*.

[19] Gao S.-M., Yang J.-J., Chen C.-Q. (2012). Pure curcumin decreases the expression of WT1 by upregulation of miR-15a and miR-16-1 in leukemic cells. *Journal of Experimental and Clinical Cancer Research*.

[20] Gebeshuber C. A., Kornauth C., Dong L. (2013). Focal segmental glomerulosclerosis is induced by microRNA-193a and its downregulation of WT1. *Nature Medicine*.

[21] Li R., Chung A. C. K., Yu X., Lan H. Y. (2014). MicroRNAs in diabetic kidney disease. *International Journal of Endocrinology*.

[22] Kato T., Mizuno S., Kamimoto M. (2010). The decreases of nephrin and nuclear WT1 in podocytes may cause albuminuria during the experimental sepsis in mice. *Biomedical Research*.

[23] Dunn K. W., Kamocka M. M., McDonald J. H. (2011). A practical guide to evaluating colocalization in biological microscopy. *American Journal of Physiology—Cell Physiology*.

[24] Sui W., Lin H., Li H., Yan Q., Chen J., Dai Y. (2014). Circulating microRNAs as potential biomarkers for nephrotic syndrome. *Iranian Journal of Kidney Diseases*.

[25] Te J. L., Dozmorov I. M., Guthridge J. M. (2010). Identification of unique MicroRNA signature associated with lupus nephritis. *PLoS ONE*.

[26] Pfaffl M. W. (2001). A new mathematical model for relative quantification in real-time RT–PCR. *Nucleic Acids Research*.

[27] Hodson E. M., Willis N. S., Craig J. C. (2007). Corticosteroid therapy for nephrotic syndrome in children. *Cochrane database of systematic reviews (Online)*.

[28] Uwaezuoke S. N. (2015). Steroid-sensitive nephrotic syndrome in children: triggers of relapse and evolving hypotheses on pathogenesis. *Italian Journal of Pediatrics*.

[29] Niaudet P., Avner E. D., Harmon W. E., Niaudet P. (2004). Steroid-resistant idiopathic nephrotic syndrome in children. *Pediatric Nephrology*.

[30] Bollain J. J., González M., Torres F. (2011). Increased excretion of urinary podocytes in lupus nephritis. *Indian Journal of Nephrology*.

[31] Ohsaki H., Sofue T., Kawakami K. (2016). WT1 immunoenzyme staining using SurePath™ processed urine cytology helps to detect kidney disease. *Cytopathology*.

[32] Barutta F., Tricarico M., Corbelli A. (2013). Urinary exosomal MicroRNAs in incipient diabetic nephropathy. *PLoS ONE*.

[33] Zhou H., Kajiyama H., Tsuji T. (2013). Urinary exosomal Wilms' tumor-1 as a potential biomarker for podocyte injury. *American Journal of Physiology—Renal Physiology*.

[34] Joshi S., Andersen R., Jespersen B., Rittig S. (2013). Genetics of steroid-resistant nephrotic syndrome: a review of mutation spectrum and suggested approach for genetic testing. *Acta Paediatrica, International Journal of Paediatrics*.

[35] Ruf R. G., Schultheiss M., Lichtenberger A. (2004). Prevalence of WT1 mutations in a large cohort of patients with steroid-resistant and steroid-sensitive nephrotic syndrome. *Kidney International*.

[36] Guo J.-K., Menke A. L., Gubler M.-C. (2002). WT1 is a key regulator of podocyte function: reduced expression levels cause crescentic glomerulonephritis and mesangial sclerosis. *Human Molecular Genetics*.

[37] Siehl J. M., Thiel E., Heufelder K. (2003). Possible regulation of Wilms' tumour gene 1 (WT1) expression by the paired box genes PAX2 and PAX8 and by the haematopoietic transcription factor GATA-1 in human acute myeloid leukaemias. *British Journal of Haematology*.

[38] Aqeilan R. I., Calin G. A., Croce C. M. (2010). MiR-15a and miR-16-1 in cancer: discovery, function and future perspectives. *Cell Death and Differentiation*.

